# *Cx3cr1*^*CreERT2*^-driven *Atg7* deletion in adult mice induces intestinal adhesion

**DOI:** 10.1186/s13041-020-00630-4

**Published:** 2020-06-08

**Authors:** Younghwan Lee, Ji-Won Lee, Hyeri Nam, Seong-Woon Yu

**Affiliations:** 1 Department of Brain and Cognitive Sciences, Daegu Gyeongbuk Institute of Science and Technology (DGIST), 333 Techno Jungang Daero, Hyeonpung-Myeon, Dalseong-Gun, Daegu, 42988 Republic of Korea; 2 Neurometabolomics Research Center, Daegu Gyeongbuk Institute of Science and Technology (DGIST), Daegu, 42988 Republic of Korea

## Abstract

Microglia are macrophages resident in the central nervous system. C-X3-C motif chemokine receptor 1 (CX3CR1) is a G_αi_-coupled seven-transmembrane protein exclusively expressed in the mononuclear phagocyte system including microglia, as well as intestinal and kidney macrophages. *Cx3cr1*^*CreERT2*^ mice express Cre recombinase in a tamoxifen-inducible manner and have been widely used to delete target genes in microglia, since microglia are long-lived cells and outlive peripheral macrophages, which continuously turn over and lose their gene modification over time. ATG7 is an E1-like enzyme that plays an essential role in two ubiquitin-like reactions, ATG12-ATG5 conjugation and LC3-lipidation in autophagy. To study the role of ATG7 in adult microglia, we generated *Cx3cr1*^*CreERT2*^:*Atg7*^*fl/fl*^ mice and deleted *Atg7* at the age of 8 weeks, and found induction of intestinal adhesion. Since intestinal adhesion is caused by excessive inflammation, these results suggest that deletion of *Atg7* in intestinal macrophages even for a short time results in inflammation that cannot be rescued by replenishment with wild-type intestinal macrophages. Our finding suggests that, depending on the roles of the gene, *Cx3cr1-Cre*-mediated gene deletion may yield unanticipated physiological outcomes outside the central nervous system, and careful necropsy is necessary to assure the microglia-specific roles of the target gene.

## Main text

Microglia are the central nervous system (CNS)-resident macrophages. Microglia are of hematopoietic origin and arise from the erythromyeloid precursor cells in the yolk sac during the early days of embryonic development [1]. Therefore, microglia are genetically and physiologically close to macrophages of peripheral tissues [1–4]. After microglia settle down in the embryonic brain, their proliferation peaks during the postnatal days 5–15 in mice [5]. Thereafter, microglia proliferate continuously but slowly; they have a long lifetime and self-renew without inflow of peripheral myeloid cells in healthy condition. On the other hand, peripheral macrophages turn over rapidly and are continuously replenished through proliferation or by macrophages newly differentiated from monocytes [6].

C-X3-C motif chemokine receptor 1 (CX3CR1) is a Gα_i_-coupled seven-transmembrane protein exclusively expressed in the mononuclear phagocyte system including microglia [7]. In adult *Cx3cr1*^*CreERT2*^ mice, Cre activity–dependent tamoxifen (TAM)-inducible gene recombination is possible in CX3CR1^+^ macrophages (microglia, intestinal, and renal) but not in CX3CR1^−^ macrophages (including peritoneal, splenic, and alveolar) [8]. Microglia are long-lived cells and outlive peripheral macrophages. Therefore, gene deletion in microglia lasts longer than in peripheral monocytes and macrophages; this unique physiological feature of microglia makes *Cx3cr1-Cre* mice an invaluable tool to express or delete genes of interest in a microglia-specific manner in the CNS [9].

Previously, we reported that Toll-like receptor activation significantly suppresses microglial autophagy flux, but stimulates autophagy in peripheral macrophages [10]. To study the physiological implications of autophagy suppression in activated microglia in the adult brain, we generated mice with an inducible microglia-targeting *Atg7* conditional knockout (cKO) mice by crossing *Atg7*^*fl/fl*^ mice with the *Cx3cr1*^*CreERT2*^ mouse line. ATG7 is an E1-like enzyme that plays an essential role in two ubiquitin-like reactions, ATG12–ATG5 conjugation and LC3 lipidation in autophagy [11]. Therefore, deletion of *Atg7* efficiently suppresses autophagy flux in vivo in the CNS as well as in vitro [12–15].

**Fig. 1:**
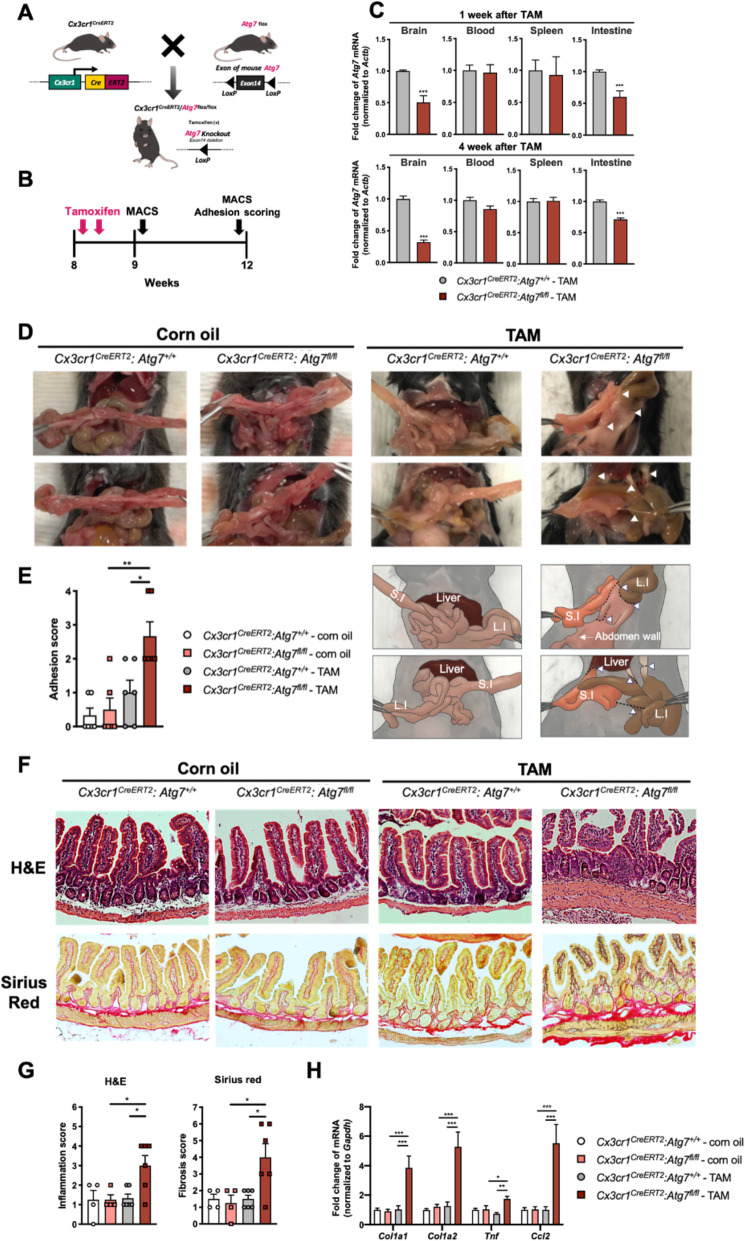
TAM injection induces intestinal adhesion in *Cx3cr1*^*CreERT2*^*:Atg7*^*fl/fl*^ mice. **a** A diagram of the generation of *Cx3cr1*^*CreERT2*^*:Atg7*^*fl/fl*^ mice. **b** Experimental schedule of MACS and intestinal adhesion scoring after TAM injection. **c** qRT-PCR analyses of the *Atg7* mRNA expression levels in microglia, PBMCs, and spleen and intestinal macrophages (*n* = 3; Mann–Whitney test, ***p < 0.001). **d** Representative images of *Cx3cr1*^*CreERT2*^*:Atg7*^*+/+*^ and *Cx3cr1*^*CreERT2*^*:Atg7*^*fl/fl*^ mice injected with corn oil or TAM. Adhesions between small (S.I) and large intestines (L.I), abdomen wall and intestines, or liver and intestines are marked with white arrowheads. The cartoon drawings below show adhesion locations. **e** Adhesion score based on Nair’s adhesion scale. TAM-injected *Cx3cr1*^*CreERT2*^*:Atg7*^*fl/fl*^ group showed a significantly higher adhesion score than the other groups (*n* = 6; one-way ANOVA test followed by Bonferroni’s multiple comparison test; F_(3,20)_ = 9.627, *p < 0.05, ** *p* < 0.01). **f** Representative images of H&E and Sirius Red stainings. TAM-injected *Cx3cr1*^*CreERT2*^*:Atg7*^*fl/fl*^ group showed infiltration of inflammatory cells (shown as blue dots) filling inside of mucosa and submucosa. The inflamed intestine also showed thickening of intestine wall. Furthermore, collagen deposition occurred in submucosa and serosa. **g** Histological scores. TAM-injected *Cx3cr1*^*CreERT2*^*:Atg7*^*fl/fl*^ group showed significantly higher inflammation and fibrosis scores than other groups (*n* = 4 for corn-oil, n = 6 for TAM; one-way ANOVA test followed by Bonferroni’s multiple comparison test; F_(3,16)_ = 5.139,, *p < 0.05). (H) qRT-PCR analyses of the inflammation and fibrosis genes. Intestine of TAM-injected *Cx3cr1*^*CreERT2*^*:Atg7*^*fl/fl*^ group showed a significantly increased gene expression (n = 3; one-way ANOVA test followed by Bonferroni’s multiple comparison test; *Col1a1,* F_(3,20)_ = 11.3; *Col1a2,* F_(3,20)_ = 15.6; *Tnf,* F_(3,20)_ = 6.88, *Ccl2,* F_(3,20)_ = 11.9, *p < 0.05, ** *p* < 0.01, ***p < 0.001)

To generate mice deficient in *Atg7* at the adult stage, 8–week-old *Cx3cr1*^*CreERT2*^:*Atg7*^+/+^ or *Cx3cr1*^*CreERT2*^:*Atg7*^fl/fl^ mice were intraperitoneally (i.p.) injected with TAM twice at a 48-h interval, and after 1 or 4 weeks, CD11b^+^ cells were isolated from blood, intestine, spleen, and brain (Fig. 1a, b). Analysis by quantitative real time-polymerase chain reaction (qRT-PCR) showed that, at 1 week after TAM injection, the *Atg7* mRNA level was reduced in microglia and intestinal macrophages isolated from *Cx3cr1*^*CreERT2*^:*Atg7*^fl/fl^ mice (Fig. 1c; Mann–Whitney test; *p <* 0.05), but not in CD11b^+^ peripheral blood mononuclear cells (PBMCs) or spleen macrophages, which originate from CX3CR1^+^ precursors but cease to express CX3CR1 at the adult stage. The *Atg7* mRNA level in microglia from *Cx3cr1*^*CreERT2*^:*Atg7*^fl/fl^ mice was further decreased at 4 weeks after TAM injection, whereas intestinal macrophages progressively recovered the *Atg7* expression (Fig. 1c). While we collected the spleens, surprisingly, we frequently found tissue adhesion in the abdomen, especially on the intestines, in TAM-injected *Cx3cr1*^*CreERT2*^:*Atg7*^fl/fl^ mice, but not in the other groups (Fig. 1d and Supplemental Movies 1, 2, 3 and 4). Indeed, quantitative analysis of the intestinal adhesion score according to Nair’s adhesion scale [16] (Table 1) indicated that TAM-injected *Cx3cr1*^*CreERT2*^:*Atg7*^fl/fl^ mice had a significantly higher score than the other groups (Fig. 1e; one-way ANOVA test; F_(3,20)_ = 9.627, *p <* 0.05).

**Table 1:** Intestinal adhesion score

Grade	Criteria for Nair’s adhesion score
0	No adhesion band
1	One filmy adhesion band between the viscera or between the viscera and the abdominal wall
2	Two thin bands between the viscera or between the viscera and the abdominal wall
3	More than two moderate bands between the viscera or between the viscera and the abdominal wall, or the whole intestine forms a mass without adhering to the abdominal wall
4	Very thick adhesion band between the viscera and the abdominal wall


**Additional file 1:Movie S1.** Representative *Cx3cr1*^*CreERT2*^*:Atg7*^*+/+*^ mouse injected with corn oil.



**Additional file 2: Movie S2.** Representative *Cx3cr1*^*CreERT2*^*:Atg7*^*+/+*^ mouse injected with TAM.



**Additional file 3: Movie S3.** Representative *Cx3cr1*^*CreERT2*^*:Atg7*^*fl/fl*^ mouse injected with corn oil.



**Additional file 4: Movie S4.** Representative of *Cx3cr1*^*CreERT2*^*:Atg7*^*fl/fl*^ mouse injected with TAM. (MP4 34858 kb)


*Atg7* or *Atg16l1* deletion is correlated with the pro-inflammatory response in the intestines [17, 18] . Deletion of *Atg7* in myeloid cells by using *LysM-Cre* mice increased susceptibility to dextran sodium sulfate–induced colitis [17]. Also, a recent report showed that ablating *Atg7* through *Cx3cr1*^*Cre*^*;Atg7*^*fl/fl*^ increased the expression of IL-23, leading to upregulation of IL-22 expression and augmented intestinal fibrosis induced by administration of 2,4,6-trinitrobenzenesulfonic acid [19], indicating that defects in autophagy contribute to intestinal inflammation when the proinflammatory cues are added. Previous examination of *Cx3cr1*^*CreERT2*^ mice showed that a TAM-induced genetic modification also occurred in intestine- and kidney-resident macrophages, which express CX3CR1 at the adult stage [8]. Since mice deficient in *Atg7* produced through *LysM-Cre* or *Cx3cr1*^*Cre*^*-*mediated deletion are susceptible to colitis and intestinal fibrosis [17, 19], our finding may also be related to the deletion of *Atg7* in CX3CR1^*+*^ intestinal macrophages.

To further test whether intestinal adhesion occurring in TAM-injected *Cx3cr1*^*CreERT2*^:*Atg7*^fl/fl^ mice was due to increased inflammation and fibrosis in the intestine, we performed hematoxyline and eosin (H&E) and Sirius Red stainings. Indeed, TAM-injected *Cx3cr1*^*CreERT2*^:*Atg7*^fl/fl^ mice with high intestinal adhesion score (≥3) clearly showed increased histological score (≥4) in both H&E and Sirius Red staining analyses (Tables 2 and 3, Fig. 1f-g). Infiltration of inflammatory cells (shown as blue dots) filled inside of mucosa and submucosa (Fig. 1f). The inflamed intestine also showed thickening of intestine wall (Fig. 1f). Furthermore, fibrotic collagen deposition occurred in submucosa and serosa in TAM-injected *Cx3cr1*^*CreERT2*^:*Atg7*^fl/fl^ mice (Fig. 1f). Concomitantly, RNA analyses for fibrosis (*Col1a1* and *Col1a2*) and inflammatory cytokines genes (*Tnf* and *Ccl2*) in whole intestine extracts also revealed marked increase in the transcript levels of the examined genes (Fig. 1h). Mild intestinal adhesion and fibrosis began from 1 week after TAM injection in *Cx3cr1*^*CreERT2*^:*Atg7*^fl/fl^ mice (data not shown).

**Table 2:** Intestinal inflammation score

Grade	Criteria for inflammation score
Inflammation	
0	Occasional inflammatory cells in the lamina propria
1	Increased number of inflammatory cells in the lamina propria
2	Confluence of inflammatory cells extending into the submucosa
3	Transmural extension of the inflammatory cells
Tissue damage	
0	No mucosal damage
1	Lymphoepithelial lesion
2	Surface mucosal erosion or focal ulceration, intestine wall thickening
3	Extensive mucosal damage and extension into deeper structure

**Table 3:** Intestinal fibrosis score

Grade	Criteria for inflammation score
Fibrosis	
0	No increase in collagen deposition
1	Increased collagen deposition in submucosa and mucosa
2	Increased collagen deposition in muscularis mucosa, submucosa, and mucosa; thickening, disorganization of the muscularis mucosa.
3	Increased collagen deposition in muscularis propria, muscularis mucosa, submucosa, and mucosa
4	Increased collagen deposition throughout all layers including serosa
Percentage	
0	0–25% of section
1	25–50% of section
2	50–75% of section
3	75–100% of section

Therefore, we assume that the intestinal phenotype observed in microglial *Atg7* cKO mice stems from this feature of the *Cx3cr1*^*CreERT2*^ system, which induces genetic recombination in both microglia and intestinal macrophages, although genetic modification is more persistent in microglia than intestinal macrophages. However, there are differences in the experimental models between the previous studies and ours. The results of the previous studies were obtained with the mouse lines in which the Cre recombinase is constitutively active and the developmental defects accumulate due to the prolonged ablation of *Atg* genes, while our study was performed in an inducible KO manner in adult mice. Also, we did not administer any disease-inducing cues, such as dextran sodium sulfate. It is possible that non-pharmaceutical-grade corn oil used to prepare TAM acted as a disease cue in *Atg7* cKO mice [20].

In conclusion, microglial *Atg7* deletion in *Cx3cr1*^*CreERT2*^*:Atg7*^*fl/fl*^ mice induced unanticipated intestinal adhesion with high frequency. *Cx3cr1*-driven, inducible and transient deletion of *Atg7* in the intestinal macrophages in adult mice offers similar phenotypes compared with the continuous deletion of *Atg7* during development into adulthood. Based on our result, we suggest to check intestinal or kidney defects in *Cx3cr1*^*CreERT2*^*:Atg7*^*fl/fl*^ mice and exclude mice with high intestinal adhesion score (≥3) from neurobehavioral tests or pathological examination of the brain. *Cx3cr1*^*CreERT2*^-mediated gene deletion may yield unanticipated physiological outcomes outside the CNS, and careful necropsy is necessary to assure the microglia-specific roles of the target gene.

## Methods

### Mice

*Cx3cr1*^*CreERT2*^ transgenic mice were purchased from The Jackson Laboratory and *Atg7*^*fl/fl*^ mice were provided by Dr. Myung-Shik Lee (Yonsei University, Korea) with the permission of Dr. Massaki Komatsu (Tokyo Metropolitan Institute of Medical Science, Japan). *Cx3cr1*^*CreERT2*^ mice were crossed with *Atg7*^*fl/fl*^ mice to generate TAM-inducible, microglia-specific *Atg7* cKO mice.

### TAM injection

TAM (Sigma, T5648) was dissolved in corn oil (Sigma, C8267) with sonication at a concentration of 20 mg/ml until particles were no longer detectable, and was stored at − 20 °C. Before injection, TAM was incubated at 37 °C with shaking overnight and 8-week-old *Cx3cr1*^*CreERT2*^:*Atg7*^+/+^ or *Cx3cr1*^*CreERT2*^:*Atg7*^fl/fl^ mice were i.p. injected with 4 mg of TAM twice with a 48-h interval. One or 4 weeks after TAM injection, mice were deeply anaesthetized by i.p. injection of Zoletil (Virbac, 6FX7; 50 mg/kg) and Rompun (Bayer; 10 mg/kg) to isolate CD11b^+^ cells from the brain, blood, spleen, and intestine. Intestine adhesion was scored 4 weeks after TAM injection following the published criteria [16, 21], as described in Table 1.

### PBMC isolation

Blood (1 ml) was collected through a peritoneal blood vessel and mixed with 15 μl of 0.5 M EDTA (Invitrogen, 15,575,020). For density gradient fractionation, whole blood was diluted with the same volume of 0.9% saline solution and overlaid on top of 15 ml Ficoll (GE Healthcare, 17–1440-02) in a 50 ml Falcon tube. The tube was centrifuged at 500×*g* at 18 °C for 10 min, and the cell layer was transferred to 1 ml of magnetic activated cell-sorting (MACS) buffer (0.5% bovine serum albumin, 2 mM EDTA in 1× phosphate buffered saline) and centrifuged at 16,000×*g* at 4 °C for 30 s. The cell pellets were resuspended in MACS buffer containing FC-γ receptor inhibitor (BD Pharmingen, 553,142; 5 μg/ml). After 10 min, cell suspensions were incubated with anti-CD11b microbeads at 4 °C for 10–20 min. CD11b^**+**^ PBMCs were isolated by using a MACS LS column (Miltenyi Biotec, 130–042-401).

### Brain microglia isolation

Brains were dissociated in the buffer (20 U/mL DNase I [Stem Cell, 07900], 0.05% collagenase D [Roche, 11,088,866,001], 15 mM HEPES, 5 mM CaCl_2_ in Hank’s balanced salt solution [HBSS; GIBCO, 14185–052]) with 0.5% glucose for 20 min at 37 °C with gentle pipetting, filtered through a 70-μm strainer, and then centrifuged at 500×*g* for 3 min. For density gradient fractionation, the pellets were suspended in 4 ml of 37% Percoll (Sigma-Aldrich, P4937) diluted in HBSS and layered over 4 ml of 70% Percoll in a 15-ml Falcon tube. Then, 4 ml of 30% Percoll was layered on top and the tube was centrifuged to form a gradient at 200×*g* at 18 °C for 40 min. After centrifugation, the cell layer was collected and used for isolation of CD11b^**+**^ cells following the same steps as for PBMCs.

### Spleen macrophage isolation

Spleens were dissociated in the buffer (20 U/mL DNase I, 0.05% collagenase D, 5 mM CaCl_2_, 5% fetal bovine serum [FBS; Hyclone, SH30084.03] in HBSS) for 20 min at 37 °C with gentle pipetting, filtered through a 70-μm strainer, and then centrifuged at 2000×*g* for 5 min. Pellets were suspended in red blood cell lysis buffer (eBioscience,00–4333-57) for 1 min to remove red blood cells, centrifuged at 2000×*g* for 3 min and suspended with MACS buffer containing FC-γ receptor inhibitor (5 μg/ml). After 10 min, cell suspensions were used for isolation of CD11b^**+**^ cells following the same steps as PBMCs.

### Intestine macrophage isolation

Intestines were dissociated in the buffer (20 U/mL DNase I, 0.1% collagenase D, 5% fetal bovine serum in HBSS) for 20 min at 37 °C with vortexing, filtered through a 100-μm strainer, and then centrifuged at 2000×*g* for 5 min. Pellets were suspended in HBSS with 5% FBS and further mechanically dissociated through a 26 G syringe. Cell suspensions were used for isolation of CD11b^**+**^ cells following the same steps as PBMCs.

### qRT-PCR

Cell pellets were lysed and RNA was isolated using an RNeasy Plus Universal Mini Kit (Qiagen, 73,404). cDNA was synthesized using the ImProm-II Reverse Transcriptase kit (Promega, A3802) and oligo-dT primers. TOPreal qPCR 2× PreMIX (SYBR Green with low ROX; Enzynomics, RT500M) containing nTaq-HOT DNA polymerase was used for qRT-PCR with a CFX96 Real-Time System (Bio-Rad). The following primers were used: *Atg7* (5′ forward: GCCAGAGGGTTCAACATGAGCA, 3′ reverse: AGCTGGAGCAGCTCATTG), *Actb* (5′ forward: AGAGGGAAATCGTGCGTGAC, 3′ reverse: CAATAGTGATGACCTGGCCGT), *Col1a1* (5′ forward: GCTCCTCTTAGGGGCCACT, 3′ reverse: CCACGTCTCACCATTGGGG), *Col1a2* (5′ forward: GTAACTTCGTGCCTAGCAACA, 3′ reverse: CCTTTGTCAGAATACTGAGCAGC), *Tnf* (5′ forward: CATCTTCTCAAAATTCGAGTGACAA, 3′ reverse: TGGGAGTAGACAAGGTACAACCC), *Ccl2* (5′ forward: AACTCTCACTGAAGCCAGCTCT, 3′ reverse: CGTTAACTGCATCTGGCTGA), and *Gapdh* (5′ forward: ATCACTGCCACCCAGAAGAC, 3′ reverse: ACACATTGGGGGTAGGAACA).

### Histological analysis

Mouse small intestines were removed and directly put in freshly prepared 4% paraformaldehyde for 24 h. Then, intestines were washed and embedded with paraffin. Embedded tissues were cut into 7 μm slices. Histological analyses of inflammation and fibrosis were performed with H&E (Abcam, ab245880) and Sirius Red staining kit (Abcam, ab150681). Inflammation and fibrosis were scored 4 weeks after TAM injection following the published criteria by using Nikon NIS-Elements [17, 22], as described in Tables 2 and 3. Summation of inflammation and tissue damage, fibrosis and percentage were designated as the inflammation score and fibrosis score, respectively.

## Data Availability

All date generated during this study are included in this article.
